# Deep Reinforcement Learning-Based Energy Consumption Optimization for Peer-to-Peer (P2P) Communication in Wireless Sensor Networks

**DOI:** 10.3390/s24051632

**Published:** 2024-03-01

**Authors:** Jinyu Yuan, Jingyi Peng, Qing Yan, Gang He, Honglin Xiang, Zili Liu

**Affiliations:** 1 School of Knowledge Based Technology and Energy, Tech University of Korea, Siheung-si 15073, Gyeonggi-do, Republic of Korea; yjy@tukorea.ac.kr; 2 China Industrial Control Systems Cyber Emergency Response Team, Beijing 100040, China; pengjingyi@cics-cert.org.cn; 3 School of Artificial Intelligence, Beijing University of Posts and Telecommunications, Beijing 100876, China; qingyan@bupt.edu.cn (Q.Y.); brianhe@bupt.edu.cn (G.H.); hlxiang@bupt.edu.cn (H.X.); 4 China Academic of Electronics and Information Technology, Beijing 100041, China

**Keywords:** wireless sensor networks, peer-to-peer communication, energy consumption, power control, deep reinforcement learning

## Abstract

The fast development of the sensors in the wireless sensor networks (WSN) brings a big challenge of low energy consumption requirements, and Peer-to-peer (P2P) communication becomes the important way to break this bottleneck. However, the interference caused by different sensors sharing the spectrum and the power limitations seriously constrains the improvement of WSN. Therefore, in this paper, we proposed a deep reinforcement learning-based energy consumption optimization for P2P communication in WSN. Specifically, P2P sensors (PUs) are considered agents to share the spectrum of authorized sensors (AUs). An authorized sensor has permission to access specific data or systems, while a P2P sensor directly communicates with other sensors without needing a central server. One involves permission, the other is direct communication between sensors. Each agent can control the power and select the resources to avoid interference. Moreover, we use a double deep Q network (DDQN) algorithm to help the agent learn more detailed features of the interference. Simulation results show that the proposed algorithm can obtain a higher performance than the deep Q network scheme and the traditional algorithm, which can effectively lower the energy consumption for P2P communication in WSN.

## 1. Introduction

### 1.1. Background Description

In recent years, the proliferation of massive intelligent sensors has led to explosive growth in emerging communication services catering to specific sensor needs [1]. However, this surge in data traffic carried by wireless sensor communication, juxtaposed with the scarcity of wireless spectrum resources, has given rise to a pressing contradiction [2]. Effectively increasing network capacity and meeting low-latency demands across different communication modes have become urgent tasks. As a key technology for future wireless communication, Wireless Sensor Network (WSN) technology leverages the anticipated characteristics of future wireless sensor communication, including ultra-high speed, ultra-large bandwidth, massive access capability, and extensive data processing [3]. By capitalizing on its core technological advantages, WSN is poised to demonstrate its significance in future wireless sensor communication applications. In recent years, communication networks such as peer-to-peer (P2P) based on Wireless Sensor Networks have garnered widespread attention [4]. This paper focuses on the typical communication scenarios of these wireless sensor networks. In future communication scenarios, applications related to entertainment, culture, and transportation, such as video streaming, will be primarily handled by sensors. This necessitates low-energy-consumption communication access to the internet or servers through cellular networks (macrocells). Additionally, critical information should be reliably and instantaneously disseminated to nearby wireless sensors through P2P communication [5]. Cooperation between nearby sensors is facilitated to support low energy consumption and low-latency information delivery. Given the stringent quality of service (QoS) requirements in communication networks, particularly in P2P communication, ensuring ultra-low latency and high reliability is crucial. For instance, the METIS project by the European Union emphasizes achieving a reliable transmission of 1600-byte data packets with a maximum end-to-end latency of 5 ms, reaching a reliability of 99.999% [6]. In practical scenarios, P2P communication often employs rational energy consumption optimization and shared spectrum resources to enhance spectrum utilization. However, this approach can lead to severe co-channel interference, consequently increasing energy consumption. Therefore, researching reasonable and effective power control and resource allocation schemes holds significant meaning.

A substantial amount of prior work has been dedicated to addressing the energy consumption optimization problem in WSN using traditional optimization methods. However, persistent challenges arise in two key aspects. On one hand, the escalating demand for various services in sensor applications necessitates the proposal of novel WSN services, such as high-reliability and low energy consumption communication. On the other hand, the optimization problem is often formulated as an NP-hard problem, making it challenging to find the optimal solution. In such cases, the optimization problem is typically decomposed into several sub-problems, each seeking local or sub-optimal solutions. Fortunately, with continuous breakthroughs and advancements in artificial intelligence technology, deep reinforcement learning (DRL) has emerged as an effective solution to address these challenges. In recent years, DRL has rapidly developed and found increasing application in communication scenarios [4]. DRL provides a robust approach to handling the WSN environment, enabling the execution of strategies in the face of uncertainties in future predictions and dynamically perceiving implicit relationships concerning latency during the communication process. Furthermore, to tackle the difficulty of finding the optimal solution, we can leverage formulas related to the ultimate goal as part of the reward mechanism. DRL agents can then explore a rational decision-making process that approaches the optimal value of the objective.

### 1.2. Related Works

WSN has emerged as a pivotal technology in shaping the future of wireless sensor communication. During this period of technological ascension, a new era of communication is poised to dominate the emerging open business markets. Each WSN sensor transcends its individuality, forming an interconnected and intelligent foundation for the era. The 3rd Generation Partnership Project (3GPP) organization has categorized WSN’s application domains into seven major classes, including security monitoring, tracking and positioning, intelligent payments, electronic healthcare, remote monitoring, smart metering, and electronic consumption. For instance, WSN can revolutionize utility meter reading processes for water and gas [7]. Replacing labor-intensive manual readings with intelligent metering, WSN enables automated data collection through network connectivity, facilitating scheduled data transmission and enabling automated detection and control. In terms of direct communication with smart sensors, WSN supports extensive data transmission, including video streaming and voice calls. Moreover, WSN can revolutionize intelligent traffic management within the Internet of Vehicles [8]. WSN sensors in vehicles collect environmental and status information, transmitting and aggregating this data to a central processor for analysis. This provides drivers with intelligent route planning, and real-time traffic updates, and even facilitates the realization of autonomous driving, enhancing urban traffic systems. Furthermore, emerging industries such as smart homes, smart agriculture, wearable sensors, and smart healthcare are experiencing rapid growth, providing sustained impetus for market and economic expansion [9].

In the field of WSN, this study focuses on typical Peer-to-Peer (P2P) scenarios, commonly used to address optimization problems. To tackle the challenges posed by changing channel information in vehicular networks, the authors in [10] investigated an end-to-end heuristic spectrum sharing approach, which alleviates the network’s demand for global channel information. In [11], the authors proposed a two-stage energy consumption optimization method. In the initial stage, each channel is assigned to a P2P pair using a general Hungarian algorithm; in the reuse stage, multiple P2P pairs are sequentially added to each channel based on channel quality and received interference prioritized. Furthermore, some work incorporates energy consumption optimization based on the sensor’s location, such as [12], which introduces two control algorithms depending on the different environments in which sensors are located. Energy consumption optimization on the shared resource pool in WSN is a hot research topic. The method proposed in [13] is centralized power control. Initially, a central controller is required, and each vehicular sensor needs to provide information, such as position, speed, and channel status, to the central controller. The central controller collects global information and then calculates the optimal control scheme considering delay constraints. The central controller transforms the problem into an optimization problem, where Quality of Service (QoS) constraints of P2P communication fall under the optimization problem’s constraints. However, finding the optimal solution in centralized approaches is often challenging, as it involves an NP-hard problem. At the same time, as the number of sensors increases, the amount of information transmitted from sensors to the central controller becomes substantial, incurring high transmission costs and computational complexity. Therefore, centralized methods are typically suitable for communication networks with a limited number of sensors. Furthermore, with the growing number of sensors, ref. [14] investigated distributed-based resource allocation and energy consumption optimization schemes. Distributed methods enhance communication resource utilization and reduce complexity.

It is noteworthy that DRL has been widely applied to address optimization problems under uncertainty [15,16]. For instance, the remarkable success of AlphaGO in recent years [17] and the superior performance of reinforcement learning in electronic games [18] have sparked interest in utilizing DRL to solve optimization problems in WSN. Substantial progress has been achieved since the introduction of DRL in the literature [19]. DRL provides a robust approach to handling the dynamic environment of WSN, allowing for strategic execution under future predictive uncertainty and the assessment of post-selection strategy impacts. This offers a promising avenue for addressing the high dynamism and large-scale challenges in WSN. Furthermore, in DRL, to tackle the difficulty of finding the optimal solution, one can leverage formulas related to the ultimate goal as part of the reward mechanism. The DRL agent can continuously experiment and make informed decisions to approximate the target value through trial and error. Importantly, DRL possesses another advantage; namely, the performance of centralized algorithms is highly limited. DRL provides conditions for distributed methods, leveraging the autonomous interaction between DRL agents and the environment. This allows them to learn independently and continually enhance decision making. Therefore, DRL opens up new avenues for addressing issues in WSN [20].

Traditional algorithms in the past often yielded local optimal solutions but overly relied on additional information such as channel conditions and interference situations, which are typically challenging to obtain perfectly, thereby limiting algorithm performance. Furthermore, with the surge in the number of intelligent sensors and the challenges posed by large-scale sensor networks, conventional energy consumption optimization algorithms struggle to adapt to this rapidly changing communication landscape. There is a strong desire to find improved wireless network energy consumption optimization solutions. The constraints of WSN networks can serve as the reward mechanism for DRL, and the combination of future rewards and immediate rewards can prevent short-sightedness. In the literature [21], the authors proposed a multi-agent problem and used Q-learning to analyze the discrete states and energy consumption optimization issues in P2P communication. However, multiple perceptual components and channel information generate a large-scale continuous state space, making Q-learning less efficient. Building upon this, the combination of Q-learning with Deep Neural Networks (DNN) addresses the challenge of large-scale continuous state space [22], while balancing authorized and P2P communication performance.

Furthermore, the paper [23] extensively discussed the energy consumption optimization problem of Deep Q Networks (DQN). Still, DQN suffers from overestimation issues. To address the drawbacks of DQN, the work in [24] was further refined in [25]. Additionally, the article [26] considers each P2P pair as an agent based on a double deep Q network (DDQN) to maximize the total capacity of authorized sensors, but it overlooks the energy efficiency of direct communication sensors. The aforementioned works seldom simultaneously consider energy consumption optimization and resource regulation, and in situations with stringent latency, they lack an analysis of intelligent agent selection.

### 1.3. Contribution of This Work

To improve the learning effect, our work employs a DRL-based approach that combines power control and resource allocation while optimizing the energy consumption of PUs. Based on DRL, this scheme is modeled as a Markov decision process (MDP), and two deep neural networks are designed to improve the learning effect, one as a training network and the other as a target network. As we know, conventional algorithms can often lead to locally optimal solutions, but they rely too much on additional information, such as channel information, interference conditions, etc., which are usually difficult to obtain perfectly, thus limiting the performance of the algorithms. Secondly, with the challenges posed by large-scale sensor networks PU to the surge in the number of smart sensors, traditional power control algorithms have been difficult to adapt to such rapidly changing communication networks. Thus, this paper proposes a multi-intelligence DRL optimization scheme, in which each P2P sensor pair is regarded as an agent, and the entire communication network multi-intelligence can adaptively control the transmission power and select spectrum resources. To solve the problem of excessive state space dimension caused by the proliferation of the number of sensors, reinforcement learning, and neural networks are combined to implement the Q-value neural network (QNN). Since the DQN algorithm has the problem of over-estimation when outputting agent actions, this paper adopts a dual QNN structure, i.e., the DDQN, to decouple action selection and target value estimation to achieve a better action selection. The rest of our paper is organized as follows: Section 2 gives an overview of WSN and DRL, Section 3 introduces the system model of P2P communication, Section 4 shows our analysis of the P2P communication and the problem formulation, Section 5 introduces the distributed algorithm based on DDQN, Section 6 introduces our experiment, and Section 7 is the conclusion.

## 2. Overview of WSN and DRL

With the development of sensor communication services, WSN-based P2P technology has become a key technology for wireless sensor communication services. We first introduce the WSN architecture under P2P communication. Then, we introduce the conceptual knowledge of DRL, which leads to the direction that needs to be studied in this paper.

### 2.1. The New Suture of WSN

In the communication network architecture of WSN, there are various intelligent sensors such as cell phone sensors, vehicular sensors, smart meters, etc. With the development of various smart mobile sensors and the rapid growth of related functions, the WSN faces challenges such as spectrum resource scarcity and severe co-channel interference. WSN communication has advantages in improving spectrum utilization and reducing communication network burden, attracting widespread attention from industry professionals. This study focuses on two communication models in WSN: the communication between sensors and the base station, and direct communication between two sensors. In addition, considering different WSNs with different characteristics, such as the impact of high-speed movement, or the changes caused by disasters, battlefields, etc., WSNs in these scenarios are very different from the WSN with P2P communication described in this paper. Therefore, the above-mentioned dynamically changing network environments may be highly likely to affect the DRL performance, which is beyond the scope of our paper’s considerations.

Compared to traditional data forwarding through the base station, P2P communication offers advantages such as increased spectrum utilization, enhanced data transfer energy consumption, and reduced latency. To improve spectrum utilization, direct P2P communication can reuse spectrum resources allocated to authorized sensors, albeit at the cost of co-channel interference. In the current scenario with a large number of sensors in use, interference issues need careful consideration, and designing link management strategies to reduce interference is crucial for enhancing communication network performance.

**Figure 1:**
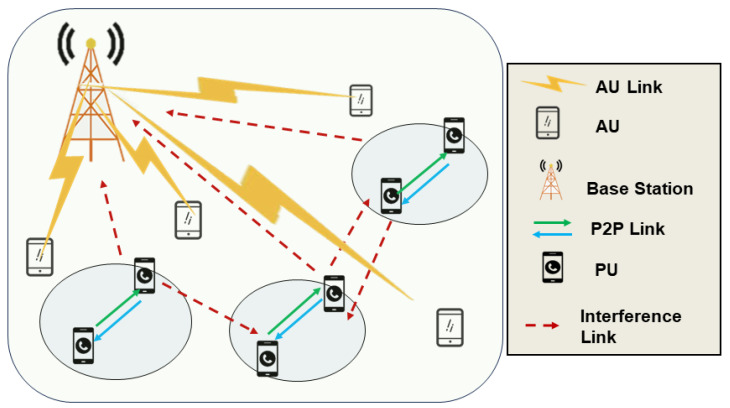
P2P communication in WSN.

In WSN communication with the identified mode of direct P2P communication reusing spectrum resources allocated to authorized sensors, link management strategies under reuse modes are crucial. Interference remains a significant factor affecting the entire communication network. The most common reuse modes are one-to-one and multiple-to-one. In a one-to-one mode, one direct P2P communication pair reuses spectrum resources allocated to one authorized sensor, ensuring better performance but with lower spectrum utilization efficiency. In a multiple-to-one mode, multiple PUs with P2P communication can reuse the spectrum resources allocated to a single authorized sensor, leading to fewer instances of idle spectrum resources. This model is the primary focus of this study. Additionally, the multiple-to-multiple mode theoretically offers greater performance gains but introduces more complex interference scenarios. Reducing interference between sensors depends on effective energy optimization and resource allocation, and a rational reuse scheme directly impacts the overall network performance. Next, we provide interference analysis for the one-to-one reuse mode. Figure 1 illustrates the communication network scene under the P2P reuse mode.

In this scenario, the P2P pair reuses the uplink of the authorized sensor. The transmission end (TX) of the direct P2P communication transmits data to the receiving end (RX), and the authorized sensor (AU) transmits data to the base station. The interference caused by TX to the base station is modeled as $I_{T,BS}=P_{T}H_{T,BS}$, where $P_{T}$ represents the transmission power of TX, and $H_{T,BS}$ represents the interference gain from TX to the base station. Simultaneously, when AU transmits data to the base station, it causes interference to RX, modeled as $I_{A,R}=P_{A}H_{A,R}$, where $P_{A}$ is the transmission power of AU, and $H_{A,R}$ represents the interference gain from AU to RX.

### 2.2. The Basic Theory of DRL

DRL is a subset of machine learning where the learning process occurs through interaction with an external environment. It is a goal-oriented learning approach, where the learner is not explicitly told how to choose actions; instead, the learner learns from the consequences of its actions. With the introduction of various related algorithms, reinforcement learning methods have undergone rapid development. In typical reinforcement learning algorithms, the steps involved are as follows:

- The agent selects an action to interact with the external environment;
- After the agent performs an action, the system transitions from the current state to another state;
- The system provides the agent with a reward based on the action taken;
- Based on the received reward, the agent learns whether the action is advantageous or detrimental;
- If the action is advantageous, meaning that the intelligent agent receives positive reinforcement, it will tend to select and execute that action. Otherwise, the intelligent agent will attempt to choose alternative actions to obtain positive reinforcement.

MDP provides a mathematical framework for solving reinforcement learning problems and is generally represented by five components:

1. Certain states that the agent can occupy;
2. Actions that the agent can choose to transition from the current state to another state;
3. Transition probabilities, representing the likelihood of transitioning from the current state to another state based on the chosen action;
4. Reward probabilities, indicating the likelihood of transitioning to another state and receiving a reward after the agent takes an action;
5. Discount factor, which alters the importance of current and future rewards and will be explained in detail later.

In typical supervised learning, machines are trained using labeled data (input and output) to generalize their knowledge, making predictions about unknown information reasonable. Supervised learning requires a supervisor with comprehensive knowledge to continuously guide the machine’s learning and correction. For example, in training a tiger to perform tricks, a trainer may specify actions such as walking forward, walking backward, or jumping through a hoop to guide the tiger explicitly. In contrast, reinforcement learning involves randomly placing a hoop in a location and letting the tiger roam freely. Each time the tiger jumps through the hoop, it receives a meat reward, and this forms the reward mechanism in reinforcement learning. The tiger continuously learns and corrects its behavior, earning more meat rewards.

In summary, the key challenge is how to judiciously apply reinforcement learning to communication scenarios, with the design of states, actions, and rewards being crucial components.

## 3. System Model

In this paper, we consider a typical P2P communication scenario as shown in the previous Figure 1. P2P sensors (PUs) multiplex the uplink spectrum resources of AUs. There are *M* AUs in the scenario, denoted as M=1,2,3,…,M; and *K* PUs, denoted as K=1,2,3,…,K. The uplink spectrum resource is divided into *F* spectrum sub-bands, denoted as F=1,2,3,…,F. Since the PUs share the spectrum resources of the AUs, the PUs cause co-channel interference to the AUs, we assume that the *f*th spectrum sub-band is allocated to the *k*th PU and use the indicator function $\varphi_{k}\left[m\right]\in \left\{0,1\right\}$ to represent the allocation decision of the PUs. In particular, $\varphi_{k}\left[m\right]=1$ if the *k*th PU reuses the spectral sub-band of the mth AU, and vice versa $\varphi_{k}\left[m\right]=0$.

The signal-to-noise-plus-ratio (SINR) of the *m*th AU is:

(1) $$\gamma^{a}\left[m\right]=\frac{P_{m}^{a}h_{m}}{\sum_{k\in K}\varphi_{k}\left[m\right]P_{k}^{p}{\hat{h}}_{k}+\sigma^{2}},$$

$P_{m}^{a}$ denotes the transmit power of the *m*th AU, $h_{m}$ denotes the channel gain of the *m*th AU, $P_{k}^{p}$ denotes the transmit power of the *k*th PU, ${\hat{h}}_{k}$ is the interference gain of the *k*th PU, and $\varphi_{k}\left[m\right]$ is an indicator for the spectrum allocation, when the *k*th PU multiplexes the spectrum of the *m*th AU, $\varphi_{k}\left[m\right]=1$, otherwise $\varphi_{k}\left[m\right]=0$, and $\delta^{2}$ denotes the noise. Based on Equation (1), we further calculate the energy consumption of *m*th AU:

(2) $$E^{a}\left[m\right]=P_{m}^{a}/\left(W\cdot \log\left(1+\gamma^{a}\left[m\right]\right)\right),$$

where *W* is the bandwidth, similarly, *k*th PU’s SINR is

(3) $$\gamma^{v}\left[k\right]=\frac{P_{k}^{p}h_{k}}{G_{a}+G_{p}+\sigma^{2}},$$

where

(4) $$G_{a}=\sum_{m\in M}\varphi_{k}\left[m\right]P_{m}^{a}{\hat{h}}_{m,k},$$

(5) Gp=∑m∈M∑k′∈Kk≠k′φkmφk′mPk′ph^k′,k,

$G_{a}$ denotes the interference introduced by the PUs to the sharing of AU spectrum resources, and if $\varphi_{k}\left[m\right]=1$, the *m*th AU will interfere with the *k*th PU. Since this paper studies the many-to-one multiplexing mode, the *k*th PU will also suffer from interference from other PUs, and $G_{p}$ denotes the interference introduced by sharing spectrum sub-bands with other PUs. If $\varphi_{k}\left[m\right]=1$ and $varphi_{k^{\prime }}\left[m\right]=1$, it means that the spectral sub-band is shared by *m*th AU, *k*thPU, and *k*th PU at the same time. $h_{k}$ denotes the channel gain of the *k*th PU, and $\hat{h_{m,k}}$ is the interference gain of the *m*th AU, and similarly, $\hat{h_{k^{\prime },k}}$ denotes the interference gain of the $k^{\prime }$th PU.

The energy consumption of the *k*th PU is

(6) $$E^{p}\left[k\right]=P_{m}^{p}/\left(W\cdot \log\left(1+\gamma^{p}\left[k\right]\right)\right).$$

Channel and interference information should be the primary observation. Assume that the instantaneous channel information of the AUs is $H_{t}\left[f\right]$, for f∈F, which represents the channel gain from the transmitter to the base station. $G_{t}\left[f\right]$, for f∈F, represents the instantaneous channel information of the PUs, and this is the interference for the AUs. $I_{t-1}\left[f\right]$, for f∈F, denotes the interference in the various spectral sub-bands in the previous moment. $N_{t-1}\left[f\right]$, for f∈F, denotes the choice of spectral sub-bands for the neighbors of the PUs at the previous moment.

## 4. Analysis and Problem Formulation

### 4.1. Analysis of P2P Communication

There are various sensor applications with different requirements in P2P networks. For example, AUs bear the bandwidth required by sensor applications or entertainment. Therefore, the requirement of AU is defined as the minimum energy consumption requirement to ensure a comfortable experience. In addition, P2P communication may be interrupted. If the interruption probability is too high, the communication quality will be very poor. P2P communication sends data information in real-time and has strict requirements on communication quality. The mathematical expressions for these related requirements are as follows:

(1) AUs and PUs energy consumption requirements: We know that links in P2P communication networks have strict quality and reliability requirements. When the constraints are met, we must lower the energy consumption of AUs and PUs as much as possible. The requirements are:

(7) $$E^{p}\left[k\right]\geq {E^{\prime \prime }}_{\min},$$

$R_{min}^{\prime }$ and $R_{min}^{\prime \prime }$ are the minimum energy consumption requirements of the network, corresponding to AUs and PUs respectively. To facilitate analysis, we assume that the requirements for all AUs and all PUs are the same.

(2) Outage probability requirement: We express the outage probability as a reliability measure. With the interruption threshold $\gamma_{0}$ and the tolerable interruption probability $P_{0}$, the reliability requirements of P2P communication are expressed as:

(8) $$P\left\{\gamma^{p}\left[k\right]\leq \gamma_{0}\right\}\leq p_{0},$$

where $\gamma^{p}\left[k\right]$ represents the signal-to-noise ratio of PUs. Under Rayleigh fading, the reliability requirement (8) can be transformed into:

(9) $$\gamma^{p}\left[k\right]\leq \gamma_{e}=\frac{\gamma_{0}}{\ln(\frac{1}{1-p_{0}})},$$

$\gamma_{e}$ is the effective outage threshold, and we again assume that the tolerable outage probability is the same for all PUs.

In our scenario, because the information on PUs is not known by the base station, the spectrum resource allocation of AUs and the spectrum resource allocation of PUs are independent of each other. Under the premise that the AUs resource allocation plan has been determined, our goal is to optimize the energy consumption of AUs and PUs. As a distributed radio resource management (RRM) scenario model, each P2P sensor needs to adjust its behavior by observing the choices of neighbor sensors. The number of neighbor sensors can be adjusted according to the situation as local information.

### 4.2. Problem Formulation

The overall goal of this paper is to find the optimal transmission power and spectrum sub-band to ensure sensor energy consumption requirements and optimize the energy consumption of AUs and PUs. The optimization problem is expressed as:

(10) $$\begin{array}{ccc} & \max_{\varphi ,p}\sum_{m\in M}E^{a}\left[m\right], & \max_{\varphi ,p}\sum_{k\in K}E^{p}\left[k\right]\\ C1: & E^{p}\left[k\right]\geq {E^{\prime \prime }}_{\min} & \\ C2: & \gamma^{p}\left[k\right]\leq \gamma_{e}=\frac{\gamma_{0}}{\ln(\frac{1}{1-p_{0}})} & \\ C3: & \varphi_{k}\left[m\right]\in \left\{0,1\right\}, & \forall k\in K,\forall m\in M\\ C4: & P_{k}^{p}\leq P_{max} & \forall k\in K \end{array},$$

where $P_{max}$ represents the maximum transmit power of PU. The optimization goal is to optimize the energy consumption of AUs and PUs. The first two constraints *C*1 and *C*2 are the energy consumption requirements for AUs and PUs. The third constraint *C*3 shows that each PU can be allocated to a spectrum sub-band, and a spectrum sub-band can be shared by multiple PUs, while the fourth constraint *C*4 indicates that the transmit power of each PU must not exceed its maximum value.

The problem formulated above is not a linear programming problem and is difficult to solve directly. The reasons are as follows. First, the resource allocation indicators $\varphi_{k}$ are all binary values, which will lead to combination problems. In addition, for AUs and PUs, the optimization objects and constraints *C*1 and *C*2 are non-convex, so the original problem has a numerical local optimal solution, and the network performance can be further improved by using the nonlinear fitting ability of the neural network. Traditional work on P2P communication mainly focuses on centralized approaches, but the acquisition of global information and large computational complexity limits their scalability to large-scale sensor communication networks. Therefore, smart distributed approaches are needed to address these challenges.

### 4.3. DRL-Based Energy Consumption for P2P Communication

For the state set sum of the P2P communication environment, the environmental state observed by each agent consists of five parts:

Instantaneous channel information of AUs, $H_{t}=H_{t}\left[1\right],H_{t}\left[2\right],\ldots ,H_{t}\left[F\right]$; instantaneous channel information of PUs $G_{t}=G_{t}\left[1\right],G_{t}\left[2\right],\ldots ,G_{t}\left[F\right]$; neighbor sensor’s sub-channel selection at the last moment $N_{t-1}=N_{t-1}\left[1\right],N_{t-1}\left[2\right],\ldots ,N_{t-1}\left[F\right]$; the interference power of each sub-channel at the previous moment $I_{t-1}=I_{t-1}\left[1\right],I_{t-1}\left[2\right],\ldots ,I_{t-1}\left[F\right]$; finally, there is the remaining load $L_{t}$, transmitted by PUs. So, the set of states are as follows:

(11) $$S_{t}=\left\{H_{t},G_{t},N_{t-1},I_{t-1},L_{t}\right\}.$$

Multi-agent: Since the algorithm in this article is distributed, each P2P treats every multi-agent as an agent. There will be multiple agents in our system. During the training process, the agent continuously interacts with the environment and then updates the weights of the neural network based on feedback.

Action: The agent chooses an action in states, This action includes the selection of transmission power and spectrum sub-bands, The transmission power is divided into four levels: Therefore, the action scale space is 4∗F, and the action space consists of transmission power and spectrum sub-bands. Define it as:

(12) $$\{sub-band_{1},\ldots ,sub-band_{F},\;power_{1},\;power_{2},\;power_{3},\;power_{4}\}.$$

Reward: When it comes to reward r, our first goal is that the agent’s actions will help lower the energy consumption of AUs and PUs. The reward function must be consistent with our goal, so the reward function consists of two parts, namely the energy consumption of AUs and the energy consumption of PUs. The sum of the energy consumptiones of AUs and PUs can directly reflect our goals and see the interference situation of the channel. The reward function is as follows:

(13) $$r_{t}=\rho_{a}\sum_{m\in M}R^{a}\left[m\right]+\rho_{p}\sum_{k\in K}R^{p}\left[k\right].$$

The discount factor can maximize rewards, not only do we have to consider current rewards, many times future rewards also determine good outcomes. We can define the formula:

(14) $$R_{t}=r_{t}+r_{t+1}+\ldots +r_{t+n}.$$

Adding the discount factor, the formula is:

(15) $$R_{t}=\sum_{n=0}^{\infty }\alpha^{n}r_{t+n}.$$

As for the final reward, the importance of the reward at each time point is not exactly the same, and the value of the discount factor is between 0 and 1. A discount factor close to 0 means that we pay more and more attention to the current reward, whereas a discount factor closer to 1 means that the current reward is less important. When the discount factor is 1, the reward function may be infinite, so the discount factor will generally not be equal to 1.

Each time the agent selects an action in states, the action includes selecting the sub-channel for transmission and the transmission power. The transmission power here is divided into three levels, so the scale of the action space is 4∗F. It should be noted that when the agent interacts with the environment, it needs to implement the ε-greedy strategy when choosing which action to perform. New actions can be explored with probability ε, or the action with the maximum value can be selected with probability 1−ε.

Q-learning can be used to achieve strategies that maximize cumulative discounted return rewards. Given the Q-value of a state, actions can be chosen to accumulate rewards, The Q-value is used to measure the quality of action in a certain state. With the Q value of the state known, we can easily choose the action:

(16) $$a_{t}=\arg\max_{a\in A}Q\left(s_{t},a\right).$$

Update formula for Q-value:

(17) $$Q_{new}\left(s_{t},a_{t}\right)=Q_{old}\left(s_{t},a_{t}\right)+\lambda \left\{r_{t}+\beta \max_{a^{\prime }\in A}Q\left(s_{t+1},a^{\prime }\right)-Q_{old}\left(s_{t},a_{t}\right)\right\}.$$

For an environment with limited states and limited actions, an exhaustive search is performed on all possible state-action pairs to find the optimal Q value. Consider an environment with many states and multiple actions in each state, such as our P2P communication scenario model, then traversing the actions in each state will take a lot of time. We often use Q-learning in small-scale, highly discrete state spaces and action spaces. To adapt to large-scale state environments, the DQN algorithm that combines DNN and reinforcement learning to become QNN is implemented. Use the nonlinear approximation capability of QNN to establish the Q table, Converting the update of the Q table into the update of the QNN weights. The network parameters can approximate the Q function, that is $Q\left(s,a,\theta \right)\approx Q^{\prime }\left(s,a\right))$. Looking back at the formula of Q-learning, $r+\beta \max Q(s^{\prime },a)$ is the target value, Q(s,a) is the predicted value, The goal is to minimize the difference between the two by learning a correct strategy. In the same way, the loss function is defined in DQN as the mean square error of the target and predicted values:

(18) $$loss=\sum_{\left(s_{t},a_{t}\right)\in D}{\left(y-Q\left(s_{t},a_{t},\theta \right)\right)}^{2}.$$

However, the problem with DQN is that the Q value is often overestimated. This is because the maximum operator is used in the learning process, and the maximum operator uses the same method for both selected actions and estimated actions. This means that when estimating the Q-values of all actions in states, the estimated Q-values will differ from the actual values PU to having some noise. PU to the influence of noise, action $a_{1}$ may have a higher Q value than the optimal action $a_{2}$. At this time, if the action with the largest Q value is selected, the suboptimal action $a_{1}$ will be taken instead of the real best action $a_{2}$. To solve this problem, this article solves this problem by setting up two independent neural networks, and the two networks learn independently. At each update, one network (the training network) is used to select a behavior, and another network (the target network) is used to act as the target behavior. Specifically, the training network will select the largest Q value after input, and return the action $a^{\prime }$ corresponding to the Q value. After input, the target network directly selects the Q value corresponding to action $a^{\prime }$ as the target value. Note that this Q value is not necessarily the largest among all output Q values of the training network. The formula for target *y* is as follows:

(19) $$y=\left\{\begin{array}{cc} r_{t}+\max_{a\in A}Q\left(s_{t+1},a,\theta \right) & inDQN\\ r_{t}+Q\left(s_{t+1},\arg\max_{a\in A}Q\left(s_{t+1},a,\theta^{-}\right),\theta^{\prime }\right) & inDDQN \end{array}\right..$$

Two neural networks decouple selection and evaluation so that overestimation problems can be avoided.

## 5. Distributed Algorithm Based on DDQN

We utilize the DDQN algorithm to solve our power control and resource allocation problems. As with most machine learning methods, you first need to train the network. We implemented an environment simulator, and the training data come from the interaction between the simulator and the agent. In the initialization scenario, the starting position of each sensor is randomly generated, AUs, PUs, and each link channel. Each sensor will calculate the distance relationship between other sensors and itself, and select sensors who are close to itself as neighbors. QNN will combine experience replay sampling. In the environment, the agent transfers from a $s_{t}$ to $s_{t+1}$ through a certain action $a_{t}$, and obtains a reward $r_{t}$. Save this transfer information ($s_{t}$, $a_{t}$, $r_{t}$,$s_{t+1}$) in a buffer called experience playback. This information is the experience of the agent.

As shown in Algorithm 1, a small batch of data is sampled to update the QNN weights, which are gradually improved as the network is updated.
**Algorithm 1:** Training process based on DDQN distributed algorithm**Input:**Environment simulator, DDQN neural network structure.1: Start:Initialize the model to generate sersors, AUs, and PUs;Random initialization of deep neural networks as a function of Q.**Cycle:**2: for epoch e=1,2,3,…,Epo3:    Generate state $S_{1}$.4:        for step t=1,2,3,…,T5:            Select the P2P pair in the system. For the agent, the action $a_{t}$            (transmission power and spectrum resources) is selected based on the policy.6:            The environment generates reward $r_{t}$ and next state $s_{t+1}$.7:            Collect experience ($s_{t}$,$a_{t}$,$r_{t}$,$s_{t+1}$) and store it in the experience pool.8:            if t=0 mod *K*9:                Generate random numbers and then sample.10:               Select the experience corresponding to the serial number to                    train the neural network θ.11:            end if12:        end for13: end for**Output: Well-trained neural network model**

## 6. Experiment Results and Evaluation

We designed a P2P communication network with a carrier frequency of 2 GHz, the road model follows the grid segment model settings, and during initialization, the sensor’s location is on the road randomly. Each sensor will observe the neighbor sensors closest to it.

The neural network is a five-layer fully connected neural network, including one input layer, one output layer, and three hidden layers, using the Relu activation function.

The learning rate is initialized to 0.001 and decreases exponentially, and the parameters in the WSN are shown in Table 1.

We compares the proposed DRL-based scheme with the other two schemes. The first is a DQN-based scheme, in which both the selection action and the evaluation action use the maximum operator operation. In the second scheme, the agent will select spectrum sub-bands in a round-robin (RR) manner.

In the RR method, we also introduce priorities based on sub-channel conditions, which is called PRR. Especially to improve the performance of PRR and random methods, we flexibly set the transmission power. We will show the results of PRR in the high-performance case.

**Figure 2:**
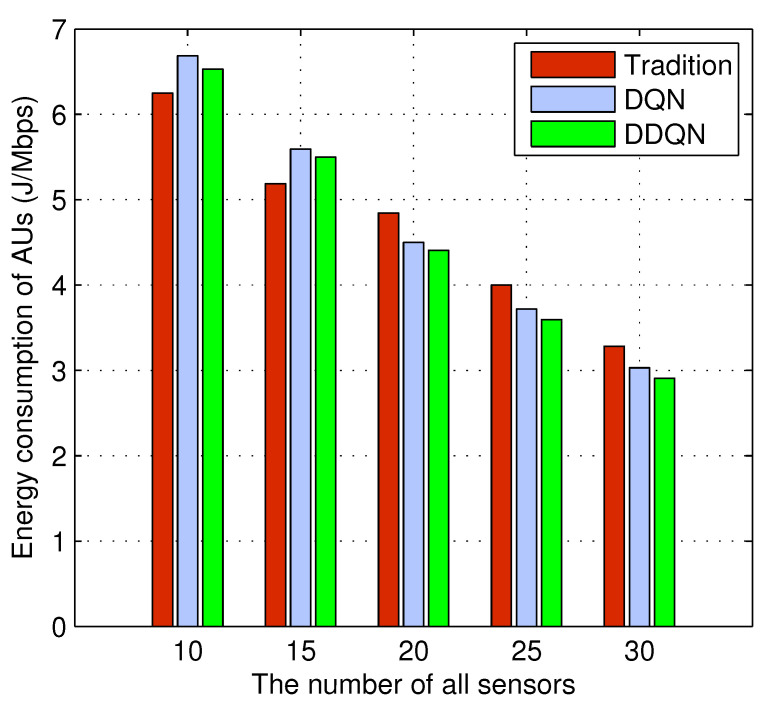
Energy consumption of AUs vs. The number of sensors under P2P communication.

Figure 2 shows the relationship between the AUs energy consumption and the number of sensors under P2P communication. First of all, it can be seen that as the number of sensors increases, the number of PUs in the network also increases. As a result, due to more and more sensors using P2P communication, the energy consumption of AUs is continuously decreasing as the number of sensors increases. Secondly, in the DRL method, DDQN shows better performance than DQN because it overcomes the disadvantage of DQN always overestimating the Q value. In addition, compared with non-reinforcement learning methods, DRL methods show overall superiority. Even if we adjust the transmission power in PRR to maximize their performance, the sensor energy consumption obtained is higher than that of the DRL method.

**Figure 3:**
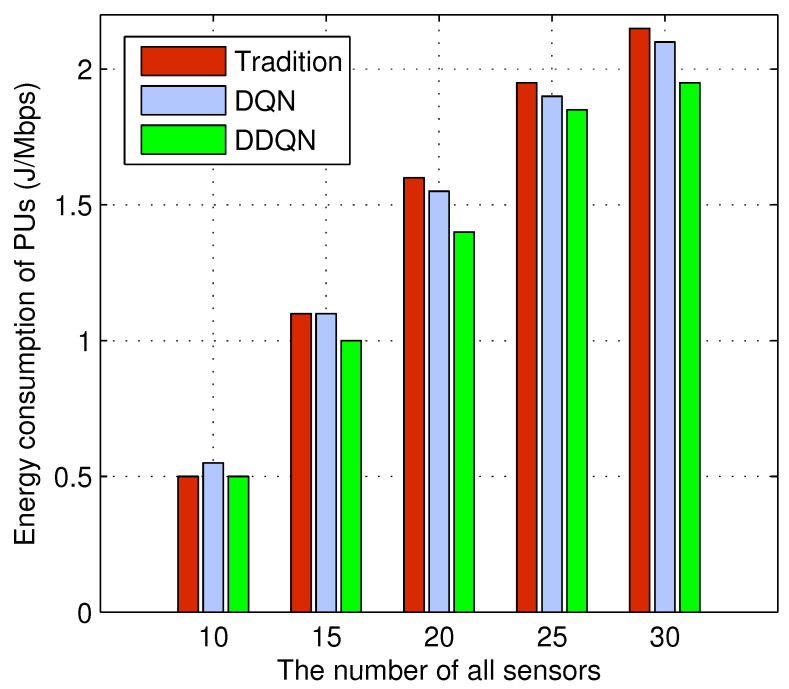
Energy consumption of PUs vs. the number of sensors under P2P communication.

Figure 3 shows the relationship between the PUs energy consumption and the number of sensors under P2P communication. First, as the number of sensors increases, the number of PUs also increases.

Therefore, the energy consumption of PUs inevitably increases. The energy consumption of PUs decreases due to more and more sensors using P2P communication. Second, as the number of sensors increases, the energy consumption gap between various methods gradually becomes larger. This is because the greater the number of sensors, the more important it is to reasonably control power and allocate resources, which reflects the advancement of DRL.

**Figure 4:**
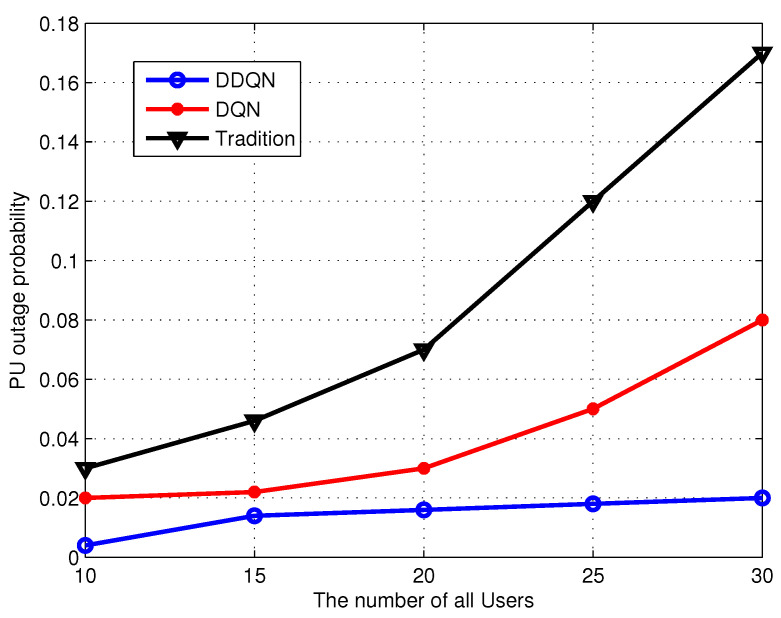
PU outage probability.

Figure 4 shows the outage probability of PUs, we can see as the number of sensors increases, the interference in the WSN becomes bigger, then, the outage probability increases. In addition, we can see DDQN has a better performance than the other two algorithms, this is PU to the agent can obtain more interference features in the networks, which can further help the agent to effectively avoid interference.

## 7. Conclusions

In this paper, we analyzed the power consumption optimization for P2P communication in the WSN. The constraint of the AU power is considered, where the proposed DDQN and DQN are proposed to optimize the energy consumption of the P2P communication. We consider the impact of the state space limitation and design a DRL-based algorithm to lower energy consumption. Last, we compare the proposed algorithm with the other two algorithms including DQN and the traditional algorithm, where we can see that our proposed algorithm can achieve the best performance.

## Figures and Tables

**Table 1:** Experimental parameters.

Parameters	Value
Noise $\delta^{2}$	−114 dBm
Reward function weights $\rho_{a}$, $\rho_{p}$, $\rho_{e}$	0.1, 0.9, 1
Maximum tolerable transmission time $T_{max}$	100 ms
P2P transmission power	30 dBm, 24 dBm, 16 dBm, 8 dBm
Carrier frequency	2 GHz

## Data Availability

Data are contained within the article.
